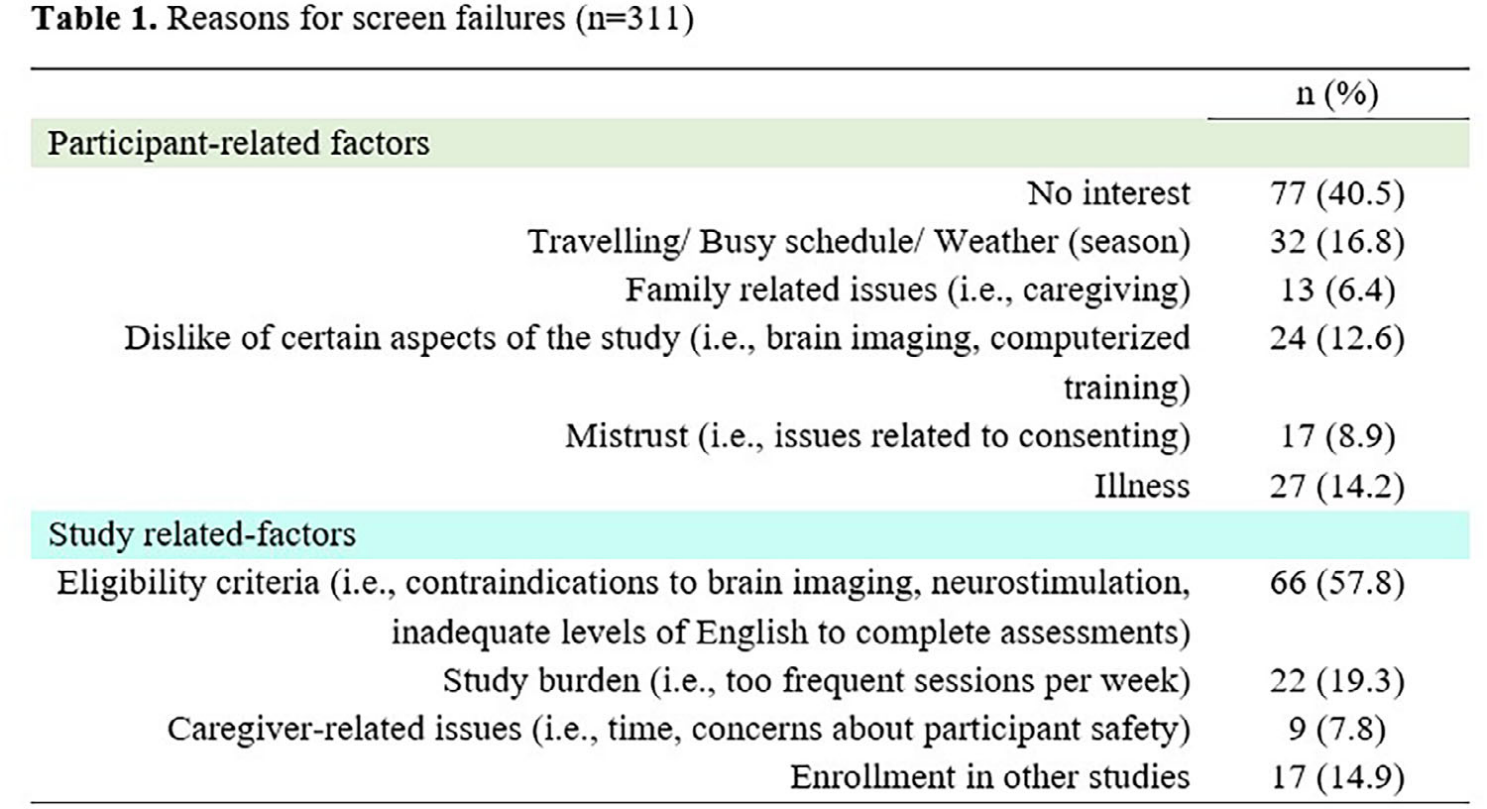# Understanding Reasons for Screen Failures in Alzheimer's disease and related dementias (ADRD) Trials: Challenges and Implications for Recruitment

**DOI:** 10.1002/alz70860_107061

**Published:** 2025-12-23

**Authors:** Oshadi M Jayakody, Ying Jin, Daniel M Aharon, David Lounsbury, Emmeline Ayers, Joe Verghese, Mirnova E Ceide, Helena M Blumen

**Affiliations:** ^1^ Albert Einstein College of Medicine, Bronx, NY, USA; ^2^ Albert Einstein College of Medicine, New York, NY, USA; ^3^ Stony Brook University, Stony Brook, NY, USA; ^4^ Stony Brook Medicine, Stony Brook, NY, USA

## Abstract

**Background:**

Recruitment challenges in ADRD clinical trials, especially when enrolling underrepresented populations, lead to significant delays, increased costs and reduced scientific merit. We aimed to examine participant‐ and study‐related barriers to recruitment and identify modifiable barriers that can be addressed in future efforts.

**Methods:**

We analyzed screen failure data from two ADRD clinical trials at the Department of Medicine, Albert Einstein College that recruited older adults from diverse populations. Two independent, blinded raters systematically reviewed screen failure cases to categorize reasons for non‐enrollment. Participant‐related barriers included lack of interest, scheduling conflicts (e.g., time/travel/weather constraints), family‐related issues, dislike of certain aspects of the study, mistrust and illness. Study‐related factors included exclusion due to eligibility criteria, study burden, caregiver‐related issues and concurrent enrollment in other studies. Disagreement between raters were resolved through discussion with a third team member. Chi‐square and Fisher's exact tests were used to determine whether participant characteristics (sex, education, ethnicity) influenced the likelihood of screen failure.

**Results:**

Data from 311 participants (mean age 78.2 (SD); 74.9% women) were analyzed. Table 1 reports the number of participants associated with each screen failure reason. Sixty two percent of screen failures were due to participant‐related factors including; no interest (40%), conflicting schedules (32%), illness (27%) and dislike of certain aspects of the study (24%). Study‐related reasons included ineligibility (57%), study burden (19%) and concurrent enrollment (14%). Notably, women reported significantly more mistrust. Among those who were screen‐failed due to eligibility criteria, 68.7% were minority older adults (Hispanic White/Black, Non‐Hispanic Black, and Asian), while 71% of those with scheduling conflicts were White older adults.

**Conclusions:**

Our preliminary findings highlight several modifiable participant‐ and study‐related barriers to target in future recruitment efforts. Participant‐related strategies could include increasing awareness of clinical trials, fostering partnerships with older adults through community programming and identifying trusted peer‐messengers. Study‐related strategies might involve carefully reconsidering eligibility criteria that may disproportionately impact diverse groups and considering additional incentives to compensate for participants’/caregivers’ time. Examining the intersectionality of social identities in relation to screen failures can provide further insights into how to improve recruitment efforts and design more inclusive ADRD clinical trials.